# Complete Cell Killing by Applying High Hydrostatic Pressure for Acellular Vascular Graft Preparation

**DOI:** 10.1155/2014/379607

**Published:** 2014-04-30

**Authors:** Atsushi Mahara, Naoki Morimoto, Takahiro Sakuma, Toshiya Fujisato, Tetsuji Yamaoka

**Affiliations:** ^1^Department of Biomedical Engineering, National Cerebral and Cardiovascular Center Research Institute, Fujishiro-dai, Suita, Osaka 565-8565, Japan; ^2^Department of Plastic and Reconstructive Surgery, Kansai Medical University, 2-5-1 Shin-machi, Hirakata City, Osaka 573-1010, Japan; ^3^Department of Biomedical Engineering, Osaka Institute of Technology, 5-16-1 Omiya, Asahi-ku, Osaka 535-8585, Japan

## Abstract

Pressure treatment has been developed in tissue engineering application. Although the tissue scaffold prepared by a ultrahydrostatic pressure treatment has been reported, an excessive pressure has a potential to disrupt a structure of extracellular matrix through protein denaturation. It is important to understand the suitable low-pressure condition and mechanisms for cell killing. In this study, cellular morphology, mitochondria activity, and membrane permeability of mammalian cells with various pressure treatments were investigated with in vitro models. When the cells were treated with a pressure of 100 MPa for 10 min, cell morphology and adherence were the same as an untreated cells. Dehydrogenase activity in mitochondria was almost the same as untreated cells. On the other hand, when the cells were treated with the pressure of more than 200 MPa, the cells did not adhere, and the dehydrogenase activity was completely suppressed. However, green fluorescence was observed in the live/dead staining images, and the cells were completely stained as red after above 500 MPa. That is, membrane permeability was disturbed with the pressure treatment of above 500 MPa. These results indicated that the pressure of 200 MPa for 10 min was enough to induce cell killing through inactivation of mitochondria activity.

## 1. Introduction

In addition to the synthetic vascular grafts made of poly(ethylene terephthalate) (PET) fibers or expanded poly(tetrafluoroethylene) (ePTFE), bioderived artificial grafts such as acellular grafts are recently commercially available. Not only the homografts (derived from human tissues) but also the xenografts are tried in the clinical stages. Decellularized whole organs are also focused on the tissue-engineered artificial organs for providing the novel treatment of organ failure [1–3].

To remove cellular fragments, the tissues were treated with the detergent such as sodium dodecyl sulfate (SDS) and washed thoroughly. Although the SDS treatment is effective for removing the cells, the remaining chemicals may be toxic, and it was reported that the repopulation on the tissue was suppressed by the detergents [4]. As alternatives, some strategies such as enzymatic treatment [5–7], hypotonic solution [8], cryochemical treatment [9], and detergent treatment [1, 9] have been reported [10].

Pressurization is the useful technology in various fields. Especially, fundamental investigation of the pressure treatment of the bacteria in food science field has been reported in several years [11, 12]. Recently, we have developed new decellularization technique by using ultrahigh hydrostatic pressure about 1000 MPa for only 10 min followed by adequate washing process in order to provide suitable tissue-engineered scaffolds that are preserving the native mechanical strength [13, 14]. In these trials, the complete cell death is of prime importance. Decellularization by the ultrahigh hydrostatic pressure (UHP) treatment does not require any toxic chemical reagents, and the cellular component can be completely washed out without any damage in the extracellular matrix (ECM). In our previous work, decellularized blood vessel was transplanted into pig descending aorta, and rapid endothelialization has been reported [13]. In these grafts, complete elimination of cellular component in addition to the sterilization effect is the critical issues in terms of the reduction of immunogenicity.

In the orthopedic surgical fields, pressure treatment has been investigated as the inactivation method of cancer cells in the tendon and bone [15–17]. In spite of several reports on pressure treatment for the decellularization and cell inactivation, the detailed effects of the pressure treatment on the cell death are not documented. In this study, we have fundamentally investigated the killing activity of the UHP treatment for mammalian cell line in order to achieve the complete cell killing or destruction with as low pressure as possible with the least protein denaturation and the other needless effects. Fibroblast, endothelial, and smooth muscle cells that were cellular components of blood vessel tissues were selected, and the effects of high pressure on cellular adhesive property, dehydrogenase activity in mitochondria, and membrane permeability were evaluated. Adhesive property of cells was evaluated by the microscopic observation 3 and 24 hrs after seeding. Dehydrogenase activity and membrane permeability of cells were evaluated by water soluble tetrazolium salts (WST) assay and live/dead staining, respectively. The results may provide the fundamental evidence of the cell death after UHP treatment of the tissue for the decellularization.

## 2. Material and Methods

### 2.1. Materials

Human umbilical vein endothelial cells (HUVECs) were cultured on tissue culture polystyrene dish (Iwaki, Tokyo, JAPAN) using endothelial basal medium (EBM; Lonza, Switzerland) supplemented with EGM SingleQuots supplements and growth factors kit (Lonza, Switzerland). Normal human aortic smooth muscle cells (SMC) were cultured with smooth muscle growth medium-2 (smGM-2; Lonza, Switzerland) supplemented with smGM2 SingleQuots supplements and growth factors kit (Lonza, Switzerland). NIH/3T3 cells were cultured on the tissue culture dish using Dulbecco's modified eagle medium (Life Technologies, Inc., Gaithersburg, MD) supplemented with 10% fetal bovine serum (FBS; MB Biomedicals, Inc., Eschwege, Germany) and antibiotics.

### 2.2. Cell Culture

The cells were grown to confluence. The cultures were placed in a humidified 95% air and 5% CO_2_ atmosphere at 37°C. The culture medium was changed every two days and confluency was typically achieved in 6–8 days. After the confluency of cells, the cells were washed with phosphate saline buffer (−) under the room temperature and immersed in 0.05% of trypsin solution containing 0.01% EDTA. After 2–5 minutes, the cells were rounded up. Trypsin was neutralized, and 2 × 10^5^ cells were seeded on 10 cm cultured dish and cultured until confluency.

### 2.3. Pressurizing of Cells

A suspension of 10^5^ cells/mL in the culture medium was packed in a plastic bag with the cell culture medium and put in a sample chamber of cold isostatic pressurization machine (Dr. Chef; Kobelco, Kobe, JAPAN) with transmission fluid. The pressure was increased up to 100, 200, 300, 500, and 980 MPa at the rate of 65.3 MPa/min and kept for 10 min. After decreasing the pressure to atmospheric pressure at the same rate, the cells were seeded into the 24-well cell culture plate (Iwaki, Tokyo, JAPAN) and cultured for 3 and 24 hours, and the morphology was observed under the microscope (Nikon TE-200; Tokyo, Japan).

### 2.4. WST-8 Assay

After the cultivation for a given period of time on 24-well plate, 10 *μ*L of WST-8 assay reagent (Dojindo, Kumamoto, Japan) was added to each well and incubated at 37°C for 1 hour. Then, the plate was gently sharked, and the absorbance at 450 nm was measured by using multiplate reader (Thermo Varioskan Flash; Thermo scientific, USA ).

### 2.5. Live/Dead Staining

UHP treated cells (4 × 10^4^ cells) were washed with PBS, and then the cells were suspended into live/dead solution which was prepared by following the provided manual (Live/Dead Cell Staining Kit II; PromoCell GmbH, Germany). The cells were incubated at 37°C for 1 hour. After the incubation, images were obtained using an Olympus FluoView confocal laser scanning microscope (Olympus, Tokyo, Japan).

### 2.6. Statistical Analysis

Quantitative results were shown as mean ± standard error of the mean. Difference between each data was evaluated by using Student's* t*-test. Significant difference was defined when *P* < 0.01.

## 3. Results

### 3.1. Cell Adhesive Property after Pressure Treatment

**Figure 1:**
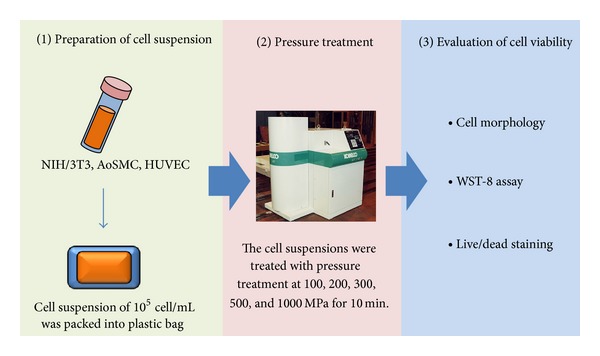
Schematic images of the evaluation of cellular viability with or without the pressure treatment. In the first step, the cell suspension was packed into the plastic bag. In the second step, the cells were treated with pressure. Finally, the cell killing was evaluated by cell morphology, WST-8 assay, and live/dead staining. The picture was cited from http://www.kobelco.co.jp/machinery/products/ip/product/cip/cip_05.html.

**Figure 2:**
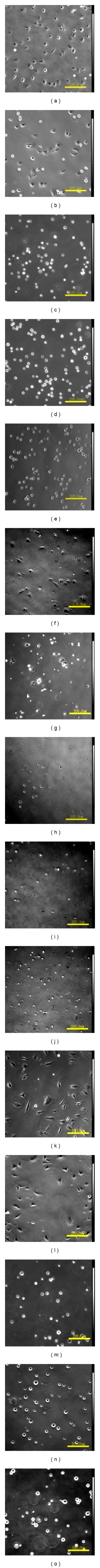
Morphology of cells after pressure treatment at 3 hours. (a–e) NIH/3T3, (f–j) smooth muscle, and (k–o) endothelial cells are shown as phase-contrast images. The cells were treated with the pressure of 0 (a, f, and k), 100 (b, g, and l), 200 (c, h, and m), 500 (d, i, and n), and 1000 (e, j, and o) for 10 min. The cells treated at 100 MPa were adhered on the culture dish as that of untreated cells.

**Figure 3:**
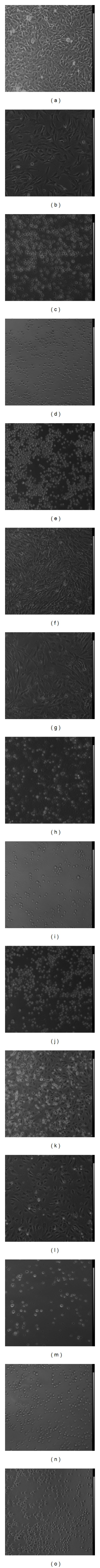
Morphology of cells after pressure treatment at 24 hours. (a–e) NIH/3T3, (f–j) smooth muscle, and (k–o) endothelial cells are shown as phase-contrast images. The cells were treated with the pressure of 0 (a, f, and k), 100 (b, g, and l), 200 (c, h, and m), 500 (d, i, and n), and 1000 (e, j, and o) for 10 min. The cells treated at 100 MPa were completely adhered on the culture dish as that of untreated cells.

The effect of pressure treatment on the cell adhesive property was evaluated by microscopic observation (Figure 1). Cells were treated at various pressures and seeded onto tissue culture plates. Cells treated with 0 and 100 MPa adhered after 3 hrs culture (Figure 2) and spread out after 24 hrs (Figure 3). Their morphologies were almost similar to the untreated cell. On the other hand, when the cells were treated with the pressure above 200 MPa, they became round shape and did not adhere on the culture dish even after 24 hours (Figure 3). The round shape morphology was not changed during the 24-hour cultivation.

### 3.2. Mitochondria Enzyme Activity of UHP Treated Cells

**Figure 4:**
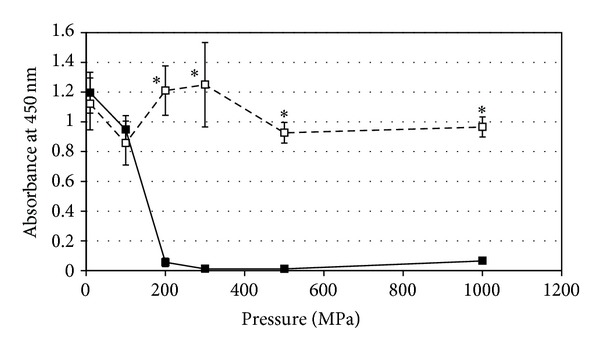
Quantification of mitochondria activity of pressure-treated NIH/3T3 cells measured by WST-8 assay. Filled square indicates the mean of the absorbance of the cells with the pressure treatment. Open square indicates the means of the absorbance without the pressure treatment as control experiments. The values shown as the mean ± standard deviation. Significant difference was identified by statistical analysis (*P* < 0.01). The mitochondria activity of the cells was completely inactivated by the pressure treatment of 200 MPa.

To evaluate the enzymatic activity in the mitochondria, WST cell viability assay was carried out. WST-8 activity against the pressure treatment is shown in Figure 4. The dehydrogenase activity in cells treated with 100 MPa was almost the same as the untreated cells. At above 200 MPa, no enzymatic activity was observed, suggesting that mitochondria enzyme was inactivated at 200 MPa. Pressure lower than 100 MPa does not affect mitochondrial enzyme activity.

### 3.3. Live/Dead Staining of UHP Treated Cells

**Figure 5:**
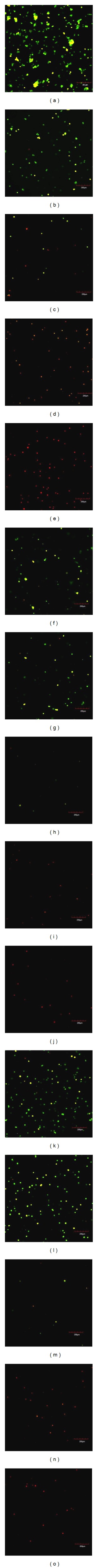
Evaluation of cell permeability by fluorescence images of UHP treated cells under live/dead staining after pressure treatment at 3 hours. (a–e) NIH/3T3, (f–j) smooth muscle, and (k–o) endothelial cells are shown as phase-contrast images. The cells were treated with the pressure of 0 (a, f, and k), 100 (b, g, and l), 200 (c, h, and m), 500 (d, i, and n), and 1000 (e, j, and o) for 10 min. The membrane permeability was disturbed with the pressure treatment of above 500 MPa.

Cell permeability and esterase activity of pressure treated cells were evaluated by CLSM images under the live/dead staining (Figure 5). The assay would mainly give the results of cell membrane damages by the pressure treatment. The green fluorescence derived from enzyme-activated calcein-AM was observed in 0, 100, and 200 MPa treated cells. After the treatment above 500 MPa, red fluorescence derived from ethidium homodimer III was mainly observed. This tendency was the same as that in each cell line. Cellular membrane was completely broken under above 500 MPa. In the condition of 200 MPa treatment, the esterase activity remained and provided the green fluorescence in the cells.

## 4. Discussion

**Figure 6:**
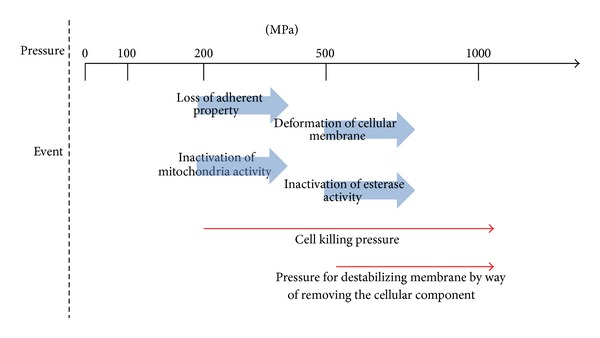
Schematic image of an effect of the pressure treatment on cell killing process. Under the pressure at below 200 MPa, the cells were adhered. When the cells were treated with the pressure at more than 200 MPa, the pressure induced the inactivation of the enzyme and cell permeability and led to cell killing.

In our previous work, the blood vessel tissue was treated with the pressure of 1000 MPa for the decellularization [13]. The cellular component was completely eliminated by the decellularization process. This study investigated the effect of the pressure treatment on cell killing. After the 100 MPa pressure treatment of cells, the cells attached on the culture dish and spread out after the seeding for 24 hours. Moreover, the dehydrogenase activity in the mitochondria was almost the same as the untreated cells. Fluorescence images of CLSM in Figure 5 were almost the same as the untreated cells after 100 MPa pressure treatment, indicating no membrane permeability. These results suggested that the 100 MPa pressure treatment did not induce the cell killing. When the cells were treated with a 200 MPa, the cells were floating on the culture dish after the cultivation at 3 and 24 hours, and the dehydrogenate activity was completely suppressed. When treated at higher 500 MPa, the cells were stained as red in live/dead staining images, suggesting that the membrane permeability largely increased. The cell killing by the pressure treatment was summarized in Figure 6. Under the low-pressure condition, the cells were alive. When the pressure was raised to around 200 MPa, the dehydrogenase activity was suppressed, and the cells were killed. When the cells were treated with higher than 500 MPa, dehydrogenase inactivation and membrane permeability destruction would synchronously occur. To induce the cell killing before washing out of cell fractions from the tissue, pressure treatment higher than 500 MPa would be beneficial for the decellularization. The cells treated at 1000 MPa were completely removed from the tissue because the decellularization was accomplished by not only the cell killing but also the deformation of cell membrane and its barrier activity.

Although large number of papers have discussed the effect of pressure treatment on bacteria, there were few reports that argue the effect on mammalian cells. In the case of mammalian cells, the sensitivity against the pressure seems to be higher than the bacteria due to the structural complexity of cells. Florian-Dominiquenaal (2005) reported that the pressure treatment of around 200 MPa induced the cell death of human chondrocytes and chondrosarcoma cells [17]. The inactivation of cellular outgrowth by pressure has been studied for the treatment of cancer therapy in orthopedic surgery [15, 16]. Mitochondria activity is largely related to an important function for cell growth such as the polymerization of actin filaments and adenine triphosphate (ADP) conversion. Therefore, 200 MPa treatment would induce the cell killing through an inactivation of mitochondria activity. Ishii et al. (2004) reported that bacterial cytoskeleton FtsZ polymers were inactivated by the pressure treatment of 40 MPa, and colony formation of* E. coli* was inhibited [18]. Although sensitivity of the pressure treatment would depend on a cell type, suppression of cytoskeleton-related enzyme activity might be directly affected by the cell killing.

Many reports discussed the effect of the pressure on bacterial cell viability defined by colony formation assay, cell wall hydrolase activity, ATP assay, and membrane potential [11, 12, 18–23]. Malone et al. (2002) reported that colony formation unit (CFU) was largely decreased by the pressure of around 200–300 MPa, and this tendency dependeds on bacteria strain [19]. The similar pressure dependency on the bacterial growth has been illustrated in many reports [12, 19, 20, 23]. The effects of pressure treatment on the membrane permeability and electric potential have also been studied [12, 19, 20, 22–24]. Malone et al. (2002) reported that cell wall hydrolase activity increased with the pressure until 400 MPa. The CFU was suppressed under the pressure of around 200 MPa, and then the deformation of membrane permeability was elicited. It is also reported that the high pressure treatment increased the cell permeability [19]. Ulmer et al. (2000) reported that the membrane activity of the bacteria was exponentially reduced, and the treatment at 500 MPa for 10 min was enough to inactivate the membrane [12]. Membrane potential also continuously decreased with increase of the pressure until 400 MPa [20, 22]. These data supported that the features of cellular membrane are largely related to the cell killing activity of the pressure treatment. However, the effect might not be a critical factor for cell killing in mammalian cells because 400–500 MPa was needed to induce a damage of the cell membrane but we found that 200 MPa is enough to kill cells. The pressure treatment decreases the metabolic and enzymatic activity [12, 19, 20, 22, 24]. The effect of esterase, ATPase, and cell wall hydrolase activities on cell growth was investigated. The pressure for inactivation of enzyme activity was largely dependent on enzyme, and the enzymatic activity was decreased during 200–400 MPa. Ishii et al. (2004) reported that the cell survival and morphology were largely correlated with the cytoskeleton polymers [18]. Therefore, the inactivation of the enzyme in mitochondria would mainly induce the cell killing.

The presented data supported that 200 MPa was enough for cell killing and 1000 MPa which we have been using for decellularization treatment was effective to remove the cells in addition to cell killing because of the enhanced membrane permeability at 500 MPa. These findings would lead us to an effective decellularization process. It is expected that detail evaluation of enzyme activity and structural analysis of the cellular component would provide significant information about the mechanisms of the cell death under the pressure treatment.

## 5. Conclusion

In this study, we suggested that the cell killing was completely induced by 200 MPa treatment through inactivation of enzyme activity in mitochondria. It is well known that the mitochondria are related to the polymerization of actin filaments and supply of the cellular energy. The pressure treatment of 200 MPa could induce the cell killing by the inactivation of mitochondria enzyme activity. On the other hand, cell membrane permeability was also changed by the pressure of more than 500 MPa. The sensitivity to the pressure would be largely related to the components of cells. In conclusion, we successfully define the effect of pressure treatment on cell killing.
